# Dietary Approaches to Stop Hypertension (DASH) Score and Its Association with Sleep Quality in a National Survey of Middle-Aged and Older Men and Women

**DOI:** 10.3390/nu12051510

**Published:** 2020-05-22

**Authors:** Hailun Liang, Hind A. Beydoun, Sharmin Hossain, Ana Maldonado, Alan B. Zonderman, Marie T. Fanelli-Kuczmarski, May A. Beydoun

**Affiliations:** 1 School of Public Administration and Policy, Renmin University, Beijing 100872, China; hliang16@jhmi.edu; 2 Department of Research Programs, Fort Belvoir Community Hospital, Fort Belvoir, VA 22060, USA; hind.a.baydoun.civ@mail.mil; 3 Health Disparities Research Section, Laboratory of Epidemiology and Population Sciences, National Institute on Aging, NIA/NIH/IRP, Baltimore, MD 21224, USA; sharmin.hossain@nih.gov (S.H.); ana.maldonado@nih.gov (A.M.); zondermana@mail.nih.gov (A.B.Z.); 4 Department of Behavioral Health and Nutrition, 206C McDowell Hall, Newark, DE 19716, USA; mfk@udel.edu

**Keywords:** the DASH diet, sleep quality, adults, national surveys

## Abstract

Complex processes appear to link sleep duration and quality with dietary patterns. Numerous studies show healthful benefits of the Dietary Approaches to Stop Hypertension (DASH) diet, but few have examined its association with sleep duration or quality. The current study tested cross-sectional associations of DASH diet quality score with sleep quality among adults. Analyses of participants were from the 2005–2008 wave of the National Health and Nutrition Examination Surveys (*n* = 3941 adults ≥30 years of age, complete data). We performed sex- and age group-stratified multiple OLS regression analyses with DASH total score and components as main predictors and sleep quality as main outcomes, adjusting sequentially for socio-demographic, behavioral and health-related factors. Sex and age differences in associations of DASH with sleep quality, adjusting for covariates, were also examined by incorporating two-way interaction terms between sex/age and the DASH score in each unstratified model. We found that the DASH diet score was inversely related to poor sleep-related daytime dysfunction adjusted by age, sex, demographic and socio-economic factors. Some sex-specific associations were detected between DASH diet component scores and sleep quality. Notably, the potassium DASH component was inversely associated with Factor 1 (“sleepiness and sleep disturbance”) among women. The fiber DASH component was associated with better sleep quality and inversely related to Factor 2 (“sleep-related daytime dysfunction”) in younger subjects. This study indicates health benefits of the DASH diet for sleep duration and quality. Future longitudinal studies and randomized placebo-controlled trials are required to ascertain protective effects.

## 1. Introduction 

Sleep is a crucial lifestyle factor that has only recently begun to draw attention in research and practice. Optimal sleep patterns have a tangible impact on chronic physical and psychological disorders [1], with poor sleep quality being related to depression, cardiometabolic disorders, cardiovascular disease and cancer incidence as well as elevated mortality rates [2,3,4,5,6,7,8]. Sleep accounts for one-third of an individual’s lifetime, with 7–8 h being the optimal daily duration for most age groups, as recommended by the National Sleep Foundation [9,10,11,12]. Biological pathways that affect the sleep–wake cycle include the circadian clock, hypothalamic neurons and hormonal factors and dark/light among many environmental signals [11,13]. Recently, a global epidemic of sleep disorders has emerged [14]. Given that those disorders are highly prevalent and carry negative impacts on general health, modifiable risk factors need to be uncovered, which can be used for prevention [1].

Complex processes appear to link sleep with dietary patterns and obesity. Emerging evidence indicates that the relationship between diet and sleep quality is bidirectional. Moreover, sleep quality may interact with dietary patterns to alter adverse health outcome trajectories.

In terms of sleep duration, studies showed that longer sleeping was linked to healthier dietary patterns that are rich in vegetables and higher in protein intake [15,16]. Similarly, good sleep quality was also associated with better diet, mostly featuring higher food diversity and lower fat intake [17,18,19]. Several mechanisms underly the relationship between diet quality and sleep patterns. For example, the intake of certain nutrients can affect hormonal pathways, altering sleep duration and quality [20,21]. Moreover, evidence suggests that a shorter sleep duration coupled with poor sleep quality can impact dietary intake, thereby increasing risks for weight-related outcomes and obesity [20,22].

Over the years, methodological developments for measuring total diet and/or dietary patterns can be categorized into two types overall, namely data-driven and dietary quality index approaches [23,24,25]. Data-driven approaches include multivariate statistical techniques including factor and cluster analyses, while the diet quality indices are usually determined by dietary recommendations or guidelines and given a fixed label. Since the dietary indices or scores are designed with the assumption that optimal dietary patterns are known and usually provide clear nutritional benchmarks, those indices have clear advantages over data-driven approaches. Additionally, dietary indices are also more easily interpretable by the general public [26].

Nowadays, the Dietary Approaches to Stop Hypertension (DASH) diet is prescribed by doctors among treatments for elevated blood pressure [27,28,29,30]. For each 2000 calories a day, DASH translates into ~6–8 daily servings of grains or grain products, ~4–5 daily servings of vegetables, ~4–5 daily fruit servings, ~2–3 daily low-fat dairy foods servings, ≤2 servings of 3 ounce of meat, poultry, or fish, ~2–3 daily servings of fats and oils, <2300 mg/day of sodium, ~4–5 weekly servings of nuts, seeds, or dry beans and <5 weekly servings of sweets [31]. DASH is considered a key component of a heart-healthy diet, as it limits sodium, saturated and trans-fat consumption, while increasing that of magnesium, potassium, calcium, fiber and proteins, thus controlling blood pressure [32,33].

Studies indicate various health benefits ascribed to the DASH diet [34,35,36], but few have examined its impact on sleep duration and quality [37]. Findings of a sleep–diet relationship in populations with distinct characteristics such as age and gender have been documented [38,39]. The objectives of the current study are to test cross-sectional associations between DASH diet scores and sleep patterns among adults participating in the NHANES 2005–2008 and to examine gender and age group differences in these associations.

## 2. Materials and Methods

### 2.1. Study Data

The NHANES consists of National Center for Health Statistics surveys that evaluate the civilian, non-institutionalized U.S. population using a health and nutritional study as well as in terms of the burden of major diseases and their risk factors [40,41]. Using stratified, multistage, cluster sampling with oversampling of specific groups [42], the NHANES collects data on demographic, socio-economic, and nutritional characteristics through in-person interviews, physical examinations and laboratory tests [43]. For the 2005–2006 wave of the NHANES, those on a low income, adolescents (12–19 years), the elderly (60+ years), and African American and Mexican American people were oversampled. For the 2007–2008 wave of the NHANES, all Hispanics, those on a low income, the elderly (60+ years) and African American people were oversampled [42]. NHANES received approval from an Institutional Review Board, whereby participants provided their informed consent. The present study’s protocol was approved by the National Institute on Environmental Health Sciences’ Institutional Review Board of the National Institutes of Health.

### 2.2. Study Sample

The NHANES has become a continuous surveillance system as of 1999. In this study, 20,497 NHANES participants were selected from the 2005–2006 (*n* = 10,348) and 2007–2008 (*n* = 10,149) waves. Individuals <30 years of age (*n* = 11,548) were excluded. We further excluded those with a history of cardiovascular or cancer diagnosis (*n* = 1969 of *N* = 8949), followed by CRP ≥10 mg/dL (*n* = 625 of *N* = 6980) total energy intake of <400 or of >5000 kcal (*n* = 988 of *N* = 6355), and death within the 1st year of follow-up (*n* = 39 of *N* = 5367). Of the remaining sample (*N* = 5328), we excluded those with missing data on dietary measures (*n* = 1387), and the remaining *N* = 3941 had complete data on sleep quality measures. Thus, the final sample consisted of 3941 adults, ≥30 years of age, with complete data on diet and sleep quality measures, in addition to all covariates, and these were retained after a series of exclusion criteria.

### 2.3. Key Outcome Measures: Sleep Quality

In the 2005–2008 NHANES, sleep-related characteristics were measured using Computer-Assisted Personal Interviews. Sleep-related items included those that tackled sleep duration, quality, disturbances and disorders. A subscale of 8 items focused on general productivity from the Functional Outcomes of Sleep Questionnaire [44] was also included. In the present study and as described elsewhere [45], we assessed several NHANES items to generate sleep quality indices. An exploratory factor analysis was conducted using 21 NHANES items, whereby higher scores reflected worse sleep quality (Supplementary Files S1 and S2). Common variances were used to extract factors and factor loadings were calculated with residual variances designated as uniqueness for each of these 21 items. This common factor model can be displayed as shown below:

$${\mathrm{Item}}_{i}\;=\sum_{j=1}^{k}\lambda_{ij}*Factor_{j}+\varphi_{i}$$

where ‘Item_i_’ is the value (1–5) of the item, ‘λ_ij_’ is the factor loading for each of these items, ‘Factor_j_’ is standardized z-score for factor j, and ‘φ_i_’ is the residual error associated with each item. Eigenvalues greater than 1 and Scree plots were used to determine the number of extracted factors that would produce the best model fit. Factor loadings were converted through varimax rotation and, subsequently, those factor loadings ≥0.40 were considered significant. Using a regression method, factor scores (*z*-scores) were predicted and adopted as markers of specific domains of sleep quality, whereby higher scores suggested poorer sleep. Once items with weak factor loadings on all factors were deleted, factor analysis using the remaining 14 items was conducted. Two factors were extracted followed by varimax rotation: **Factor 1** (“Sleepiness and sleep disturbance”) and **Factor 2** (“Poor sleep-related daytime dysfunction”) (Supplementary File S2).

### 2.4. Dietary Assessment and the DASH Diet

Two waves of the NHANES (2005–2006 and 2007–2008) included dietary components consisting of two 24 h recalls that were administered by trained Mobile Examination Center (MEC) interviewers. The Computerized Automated Multiple Pass Method by the U.S. Department of Agriculture (USDA) was used to collect dietary intake data [46]. The 1st dietary recall interview was collected in person in the MEC and the 2nd interview was collected by telephone 3–10 days afterwards. Nutrient intakes were later estimated by linking dietary intake with the USDA’s Food and Nutrient Database for Dietary Studies database [47,48]. Average daily nutrient intake from two 24 h recalls was used in this analysis.

In this study, the DASH diet score was determined for each participant using the Mellen et al. formula [47]. The DASH score is divided into 9 target nutrients (total fat, saturated fat, protein, fiber, cholesterol, calcium, magnesium, sodium and potassium). Micronutrient goals were reported per 1000 kcal. The total DASH score was calculated as the sum of nutrient targets met: a value of 1 was given if the participant achieved a DASH target for a nutrient, a value of 0.5 was given if an intermediate target was achieved and a value of zero was given if neither target was met Supplementary File S3. Given that previous studies have reported associations between individual nutrients that constitute the components of the Mellen DASH score [16,22,49,50,51] and sleep, we have examined those components in addition to the total DASH score in relation to sleep indices. Thus, each of the nine components of the DASH total score were considered as separate exposures in this study, in addition to the total score.

### 2.5. Covariates

Socio-demographic characteristics were defined as follows: age (in y), sex (M/F), race (Hispanic: Mexican American, Hispanic: Other, Non-Hispanic White, Non-Hispanic Black, and Other), level of education (<HS, 9–11th Grade, HS or GED equivalent, some college or Associate’s degree, ≥College Graduate), marital status (married and/or living with partner vs. other) and poverty status as indicated by the poverty-income ratio [PIR] (<100%, ≥100% to <200% and ≥200%). Lifestyle characteristics were focused on smoking status (non-smoker vs. ex-smoker vs. current smoker), alcohol consumption ≥12 glasses (in past 12 months) [Y/N], and physical activity in the past 30 days (walking/bicycling, tasks around home/yard, moderate activity or vigorous activity) [Y/N]. Lastly, health characteristics were defined by body mass index (BMI) categories, and self-rated health (SRH). BMI was calculated as weight (kg) divided by height squared in m^2^. We used the World Health Organization definition of weight status to categorize BMI as under/normal weight (BMI: <25 kg/m^2^), overweight (BMI: 25–29.9 kg/m^2^), and obese (BMI ≥30 kg/m^2^). A general health questionnaire item, “Would you say your health in general is- excellent, very good, good, fair or poor?” was used to define SRH, and this variable was further dichotomized as “excellent/very good/good” vs. “fair/poor”.

Allostatic load was determined using several variables from the 2005–2006 and 2007–2008 NHANES waves. It was previously defined as an “outcome of cumulative effects of repeated or chronic exposure to chemical and non-chemical stressors that result in a shift from a normal to an adaptive but dysfunctional state which can negatively impact physical and mental health” [52,53]. As proposed by Thomson and colleagues [52], an allostatic load index (ALI) was calculated as a composite score ranging between 0 and 9 using several biomarkers (systolic blood pressure, diastolic blood pressure, resting heart rate, high sensitivity C-reactive protein (hsCRP), serum albumin, total cholesterol, high-density lipoprotein (HDL), glycated hemoglobin (HbA1c), and finally waist-to-hip ratio). A higher ALI score would generally indicate worse allostatic load. In the absence of anthropometric measures of hip circumference from the 2001–2010 waves of NHANES, we adopted the ALI definition as described by Rodriquez and colleagues [53]. The nine components of ALI with cutoffs are found in Supplementary File S4.

### 2.6. Data Handling and Statistical Analysis

Statistical analyses were performed using Stata 16.0 [54]. We estimated variances using Taylor series linearization taking into consideration the sampling design complexity. MEC exam weights were incorporated in the analysis accounting for unequal probability of sampling for certain population groups and to obtain population estimates of means, proportions and regression coefficients [55]. This was done by using Stata survey commands and specifying weights, strata and primary sampling units (PSU) [56].

Initially, we examined differences in continuous and categorical measures across gender by design-based F-tests (*svy*:tab and *svy*:reg, respectively). Second, we conducted gender-stratified multiple OLS regression models with DASH total score as the main predictor and sleep quality measures as the main outcomes, adjusting sequentially for socio-demographic, lifestyle and health-related factors: Model 1 (age); Model 2 (age, sex, race/ethnicity, marital status, poverty income ratio and education); Model 3 (Model 2 + smoking, alcohol use, physical activity, self-rated health, BMI and ALI). Similar models were examined with components of the DASH score as main predictors. Sex differences in the associations between DASH and sleep quality measures, adjusting for covariates, were tested by adding two-way interaction terms between gender and the DASH score in each unstratified model, with the main effect of gender included.

Given missing data, particularly in sleep measures, we constructed two-stage Heckman selection models [57,58] to account for potential selection bias in all main analyses following methods applied previously in a similar sample [59].

All p-values presented were two tailed, whereby *p* < 0.05 was considered statistically significant and *p* < 0.10 was considered marginally significant.

## 3. Results

### 3.1. Study Participant Characteristics by Gender and Age Group

Table 1 and Table S1 describe study participant characteristics across gender and age groups, respectively. Among key findings, DASH diet total score and some of its components were significantly higher among women and the older group, compared to men and the younger group, respectively. While there were no racial/ethnic or socio-economic distributional differences by gender, women were less likely to be married, to be current or former smokers, and to be alcohol users, compared with men; they also had lower BMI and ALI. The percent of women, NH whites and SES group with 100% to less than 200% poverty among the older age group exceeded that within the younger age group. The older group had poorer self-rated health, higher ALI, a lower likelihood of reporting physical activity, of consuming alcoholic beverages, of being currently married or partnered, or being a current smoker compared to the younger group.

### 3.2. DASH Diet Total Score and Sleep Quality by Gender and Age Group: Multiple Linear Regression Models

Table 2 and Table S2 test the main hypothesis of an association between DASH diet total score and sleep quality Factors 1 and 2, stratifying by gender and by age group. In models adjusted only by age and sex (Model 1, *β* = −0.021, *p* = 0.028) or additionally adjusted for socio-demographic and SES factors (Model 2: *β* = −0.021, *p* = 0.038), DASH diet score was inversely associated with poor sleep-related daytime dysfunction in the total population. Nevertheless, after fully adjusting for lifestyle and health-related factors, this association was appreciably attenuated (*β* = −0.017, *P* = 0.10). The results were largely homogenous by gender and by age group.

### 3.3. DASH Diet Component Scores and Sleep Quality by Gender and Age Group: Multiple Linear Regression Models

Using a similar multiple regression modeling strategy, Table 3 and Table S3 show associations between DASH diet component scores and each of the sleep quality extracted factors. Despite homogeneity in associations, some gender-specific associations were detected. Most notably, among women, and even after full adjustment for potential confounders, the potassium component of DASH was inversely associated with sleepiness and sleep disturbance (Factor 1) among women. A similar pattern was observed in women for Factors 1 and 2 and the fiber component of DASH (Model 1 only), and for Factor 2 vs. the magnesium component of DASH (Model 2 only). Age group-stratified findings indicated that the fiber component of the DASH was associated with better sleep quality by being inversely related to Factor 2 in the younger group.

However, a reverse pattern of association, though only marginally significant (*p* < 0.10), was found in the older adults group, with a significant DASH by age group interaction (*p* < 0.05) in the three models.

## 4. Discussion

### 4.1. Main Findings

The current study investigated associations of DASH score with quality of sleep in a national survey of middle-aged and older men and women. A higher DASH diet quality score was inversely associated with poor sleep-related daytime dysfunction in the overall population. This association was observed after adjusting for socio-demographic and SES factors, as well as age and sex, and some gender-specific associations were detected between DASH diet component scores and sleep quality. Most notably, the potassium component of DASH was inversely associated with sleepiness and sleep disturbance among women. The association of the DASH fiber component with better sleep quality was attributed to the inverse relationship to Factor 2 in the younger group.

### 4.2. Previous Studies

Among this study sample, high dietary quality, measured by DASH scores, was associated with better sleep quality. Other researchers have found similar results despite using different diet quality indices, specifically the Alternative Healthy Eating Index-2010 [16], the Healthy Nordic Food Index [60] and the Mediterranean Diet score [49]. Among a US Hispanic sample, people who slept less than 6 h nightly had significantly lower diet quality [16].

The study findings provide evidence that in women, high intakes of potassium, magnesium and dietary fiber enhance sleep quality. Additionally, among those aged 30–59 years, dietary fiber was associated with better quality of sleep. Grandner and colleagues conducted a cross-sectional study which also found an association of dietary fiber and sleep. They reported that low intake of dietary fiber was associated with indicators reflective of poor sleep quality at a higher frequency [51]. Our findings are consistent with reports that higher intake of fruits and vegetables, foods rich in potassium, magnesium, and dietary fiber, were associated with sleep duration and quality [22,49,50]. Significantly lower intakes (per 1000 kcal) of potassium, dietary fiber, and lower intakes of vegetables and nuts and legumes were reported for Hispanic adults with short sleep duration (defined as <6 h per night), compared to other sleepers [16]. Ikonte et al. reported that participants in the NHANES 2005–2016 who slept ≤7 h per night had lower usual intakes of magnesium compared to all adults, 19+ years, even after adjusting for all covariates. In both young (19–50 y) and older (51–99 y) women, they also found short sleep duration to be associated with prevalence of inadequate intakes of magnesium and 10 other micronutrients [61].

The association of magnesium with sleep has been explored by other investigators [20,51]. Magnesium appears to have a role in sleep–wake cycles and act as a cell-autonomous timekeeping component [62,63]. It also plays a role in melatonin synthesis [64]. However, the specific mechanisms by which magnesium aids sleep are unknown.

### 4.3. Biological Plausibility

The DASH diet is rich in proteins, antioxidants and plenty of micronutrients that improve the functioning of body systems. It is possible that sleep quality and duration are influenced by tryptophan, the amino acid that synthesizes serotonin, a sleep-inducing agent. A high protein diet may improve the availability of tryptophan, and therefore increase serotonin and improve sleep quality [65]. Moreover, the tyrosine metabolism pathway and metabolites in the phenylalanine were also associated with the duration of sleep [66]. Additionally, sleep quality has been positively associated with individual lipids and lipid metabolic pathways. The metabolite of furan fatty acids was found to be positively associated with sleep mid-point by Xu and colleagues [67]. Food sources of furan fatty acids include fish, butter, fruits and vegetables. These fatty acids are also derived from gut microbiota [68]. Although the DASH sodium component was not significant in this study, it is recognized that high-salt diets can affect sleep quality. Xie et al. found that young *Drosophila* on high-salt diet exhibit a fragmented sleep phenotype similar to that of normal aging individuals [69]. The induced sleep changes were attributed to impaired circadian rhythms and were dopaminergic system dependent [69]. It is worth noting that approximately 10% of our selected sample were hypertensive with systolic blood pressure ≥150 mm Hg and/or diastolic blood pressure ≥90 mm Hg (7.2% in the 30–59 yo group; 20.1% in the 60+ yo group, *P* < 0.05, Wald test for age group difference), with no gender differences.

### 4.4. Strengths and Limitations

This study has four strengths. First, the current study is nationally representative, based on a large population-based cohort. Second, our study not only investigates the association of DASH score with sleep quality, but also stratifies the results by sex and age. Third, this study includes the whole group of the NHANES rather than only including a subgroup of the population, such as the elderly or a region-wide population, which improves external validity over previous studies. Fourth, we used objective measures rather than self-reported measures for clinical characteristics, limiting recall bias compared to prior studies. Lastly, compared to previous survey-based studies, sleep indices used in this study were generated by factor analysis, which improves the validity of the sleep construct.

Since there are study limitations, the findings should be interpreted in light of these limitations. First, our study cannot establish temporal relationships between the exposure and outcome variables, since the DASH diet and sleep behaviors were measured simultaneously. Second, the secondary analysis of existing data limits our ability to ascertain certain characteristics and potentially leads to misclassification bias. Third, many of the covariates can be considered as time varying; however, the current study could only assess those covariates once in the absence of repeated measurements within the NHANES. Fourth, our study findings can only be generalized to U.S. adults aged 30 years and above, and more evidence is required to confirm these findings in a wider population.

## 5. Conclusions

The DASH diet is considered a key component of a heart-healthy lifestyle, which emphasizes the right portion sizes, a variety of foods and nutrients. Many previous studies show wide-ranging health benefits of the DASH diet, but research concerning its impact on sleep duration and quality is scarce. To address this gap, our study found that the DASH diet was inversely related to poor sleep quality. According to the National Sleep Foundation, sleep quality among the U.S. population has been and continues, to the present day, to be poor [70]. Protective lifestyle factors, such as a healthy diet and regular physical exercise, have positive associations with better sleep quality, pointing out that strategies aiming to enhance healthy food intake and dietary patterns should be implemented. This study found that the DASH diet shows promise as a sleep modulator, but additional research is needed to draw definitive conclusions. Future studies should include larger samples, encompassing both adults and children, and should also focus on people with sleep disorders.

## Figures and Tables

**Table 1:** Study sample characteristics by gender, the NHANES 2005–2008.

	Men			Women			P_gender_
	N	Mean/%	(SE)	N	Mean/%	(SE)	
Age (years):							
Mean ± SEM		48.5	0.5		50.1	0.5	<0.001
Race/Ethnicity:							0.28
Mexican American		7.6	0.01		6.7	0.01	
Other Hispanic		3.6	0.01		3.6	0.01	
Non-Hispanic White		75.9	0.02		75.1	0.02	
Non-Hispanic Black		8.1	0.01		9.6	0.01	
Other		4.8	0.01		5.0	0.01	
Education:							0.25
<Less Than 9th Grade		5.4	0.01		4.9	0.01	
9–11th Grade		10.7	0.01		11.4	0.01	
High School Grad/GED or Equivalent		24.6	0.01		23.9	0.01	
Some College or AA Degree		28.1	0.01		31.0	0.01	
College Graduate or Above		31.2	0.02		28.8	0.02	
Marital Status:							<0.001
Married/Living with Partner		77.8	0.02		67.5	0.02	
Other		22.2	0.02		32.5	0.02	
Poverty-Income Ratio:							0.11
<100%		8.0	0.01		9.5	0.01	
100%–<200%		16.2	0.01		17.0	0.01	
≥200%		75.8	0.02		73.5	0.02	
Smoking Status:							<0.001
Never Smoker		47.5	0.02		57.8	0.01	
Ex-Smoker		29.7	0.01		23.2	0.01	
Current Smoker		22.8	0.01		19.0	0.01	
Alcohol Consumption (≥12 glasses in past 12 months):							<0.001
Yes		85.8	0.01		67.3	0.02	
No		14.2	0.01		32.7	0.02	
Physical Activity:							0.062
Yes		46.3	0.03		42.2	0.03	
No		53.7	0.03		57.8	0.03	
Body Mass Index (kg/m^2^):							
Mean ± SEM		29.1	0.18		28.6	0.25	0.012
Self-Rated Health:							0.74
Excellent/Very Good/Good		87.0	0.0		87.0	0.01	
Fair/Poor		13.0	0.0		12.6	0.01	
Allostatic load							
Mean ± SEM		3.08	0.04		2.61	0.05	<0.001
DASH Diet Total Score							
Mean ± SEM		1.93	0.04		2.36	0.05	<0.001
DASH Diet Component 1: Saturated Fat							
Mean ± SEM		0.254	0.007		0.260	0.011	0.55
DASH Diet Component 2: Total Fat							
Mean ± SEM		0.265	0.009		0.274	0.009	0.51
DASH Diet Component 3: Protein							
Mean ± SEM		0.354	0.012		0.342	0.013	0.34
DASH Diet Component 4: Cholesterol							
Mean ± SEM		0.245	0.009		0.290	0.010	0.001
DASH Diet Component 5: Fiber							
Mean ± SEM		0.121	0.010		0.205	0.011	<0.001
DASH Diet Component 6: Magnesium							
Mean ± SEM		0.142	0.008		0.243	0.011	<0.001
DASH Diet Component 7: Calcium							
Mean ± SEM		0.308	0.009		0.437	0.012	<0.001
DASH Diet Component 8: Potassium							
Mean ± SEM		0.106	0.005		0.202	0.009	<0.001
DASH Diet Component 9: Sodium							
Mean ± SEM		0.134	0.008		0.113	0.006	0.040
Sleep Quality Measures							
Factor 1: Sleepiness and Sleep Disturbance							
Mean ± SEM		−0.059	0.026		+0.161	0.024	<0.001
Factor 2: Poor Sleep-Related Daytime Dysfunction							
Mean ± SEM		+0.013	0.023		+0.041	0.024	0.44

Abbreviations: DASH—Dietary Approaches to Stop Hypertension; nc—not computed; NHANES—National Health and Nutrition Examination Surveys; SEM—Standard Error of the Mean. *N*—1922 among men and *N*—2019 among women.

**Table 2:** DASH total score as a predictor of sleep quality measures, stratified by gender: multiple linear regression models, the NHANES 2005–2008.

	Total			Men			Women			
	*N* = 3941			*N* = 1922			*N* = 2019			
	Factor1: Sleepiness and sleep disturbance			Factor 1: Sleepiness and sleep disturbance			Factor 1: Sleepiness and sleep disturbance			
	β	(SEE)	P_wald_	β	(SEE)	P_wald_	β	(SEE)	P_wald_	P_gender × DASH_
Model 1	−0.025	0.0146	0.093	−0.014	0.024	0.547	−0.033	0.016	0.042	0.47
Model 2	−0.016	0.0150	0.299	−0.002	0.025	0.941	−0.024	0.015	0.115	0.36
Model 3	−0.012	0.0150	0.426	+0.001	0.026	0.969	−0.020	0.014	0.169	0.37
	Factor 2: Poor sleep-related daytime dysfunction			Factor 2: Poor sleep-related daytime dysfunction			Factor 2: Poor sleep-related daytime dysfunction			
	β	(SEE)	P_wald_	β	(SEE)	P_wald_	β	(SEE)	P_wald_	P_gender × DASH_
Model 1	−0.023	0.010	0.028	−0.027	0.014	0.068	−0.019	0.013	0.132	0.74
Model 2	−0.021	0.010	0.038	−0.022	0.015	0.149	−0.018	0.011	0.131	0.84
Model 3	−0.017	0.010	0.106	−0.019	0.016	0.249	−0.013	0.011	0.240	0.78

Abbreviations: DASH—Dietary Approaches to Stop Hypertension; NHANES—National Health and Nutrition Examination Surveys; SEE—standard error of the estimate. Model 1 (age); Model 2 (age, sex, race/ethnicity, marital status, poverty income ratio and education); Model 3 (Model 2 + smoking, alcohol use, physical activity, self-rated health, BMI and ALI).

**Table 3:** DASH component scores as predictors of sleep quality measures, stratified by gender multiple linear regression models, the NHANES 2005–2008.

	Men			Women			
	Factor 1: Sleepiness and Sleep Disturbance			Factor 1: Sleepiness and Sleep Disturbance			
	β	(SEE)	P_wald_	β	(SEE)	P_wald_	P_gender × DASH_
DASH component 1: Saturated fat							
Model 1	−0.089	0.092	0.34	−0.014	0.089	0.12	0.61
Model 2	−0.022	0.095	0.82	−0.085	0.089	0.35	0.64
Model 3	−0.014	0.097	0.89	−0.079	0.084	0.36	0.59
DASH component 2: Fat							
Model 1	+0.050	0.087	0.57	−0.011	0.066	0.10	0.17
Model 2	+0.079	0.086	0.37	−0.085	0.065	0.20	0.17
Model 3	+0.084	0.089	0.35	−0.079	0.066	0.24	0.17
DASH component 3: Protein							
Model 1	−0.033	0.050	0.51	−0.063	0.055	0.26	0.70
Model 2	−0.013	0.050	0.80	−0.053	0.052	0.32	0.57
Model 3	−0.128	0.051	0.80	−0.056	0.052	0.30	0.60
DASH component 4: Cholesterol							
Model 1	+0.014	0.072	0.84	+0.024	0.059	0.70	0.93
Model 2	+0.082	0.074	0.91	+0.024	0.060	0.69	0.83
Model 3	+0.011	0.078	0.89	+0.024	0.059	0.68	0.89
DASH component 5: Fiber							
Model 1	−0.180	0.117	0.13	−0.190	0.092	0.046	0.93
Model 2	−0.100	0.122	0.40	−0.144	0.094	0.136	0.70
Model 3	−0.087	0.123	0.49	−0.125	0.096	0.203	0.72
DASH component 6: Magnesium							
Model 1	0.035	0.123	0.78	-0.011	0.080	0.16	0.31
Model 2	0.096	0.122	0.43	−0.079	0.076	0.31	0.19
Model 3	0.111	0.125	0.39	−0.061	0.076	0.42	0.20
DASH component 7: Calcium							
Model 1	−0.081	0.074	0.28	−0.073	0.051	0.89	0.39
Model 2	−0.085	0.083	0.31	+0.018	0.048	0.97	0.39
Model 3	−0.070	0.080	0.39	+0.017	0.047	0.72	0.41
DASH component 8: Potassium							
Model 1	−0.010	0.139	0.48	−0.240	0.089	0.013	0.34
Model 2	−0.080	0.134	0.56	−0.220	0.089	0.018	0.24
Model 3	−0.068	0.135	0.62	−0.190	0.085	0.031	0.30
DASH component 9: Sodium							
Model 1	0.023	0.098	0.82	0.069	0.077	0.38	0.70
Model 2	0.043	0.092	0.65	0.088	0.075	0.25	0.70
Model 3	0.036	0.099	0.72	0.095	0.074	0.21	0.65
	**Factor 2: Poor sleep-related daytime dysfunction**			**Factor 2: Poor sleep-related daytime dysfunction**			
DASH component 1: Saturated fat							
Model 1	−0.013	0.061	0.039	−0.062	0.072	0.40	0.48
Model 2	−0.098	0.064	0.137	−0.065	0.072	0.38	0.53
Model 3	−0.088	0.069	0.209	−0.045	0.075	0.56	0.50
DASH component 2: Fat							
Model 1	−0.070	0.051	0.18	−0.046	0.060	0.45	0.78
Model 2	−0.043	0.048	0.37	−0.049	0.062	0.44	0.84
Model 3	−0.040	0.047	0.47	−0.038	0.061	0.54	0.87
DASH component 3: Protein							
Model 1	−0.067	0.056	0.24	−0.025	0.057	0.66	0.55
Model 2	−0.059	0.056	0.30	−0.022	0.056	0.69	0.55
Model 3	−0.066	0.054	0.23	−0.034	0.054	0.53	0.65
DASH component 4: Cholesterol							
Model 1	−0.011	0.062	0.087	−0.023	0.052	0.66	0.33
Model 2	−0.012	0.061	0.057	−0.018	0.051	0.74	0.30
Model 3	−0.012	0.060	0.060	−0.010	0.052	0.84	0.22
DASH component 5: Fiber							
Model 1	+0.042	0.076	0.59	−0.140	0.068	0.049	0.26
Model 2	−0.013	0.075	0.87	−0.120	0.066	0.085	0.23
Model 3	+0.002	0.076	0.98	−0.090	0.071	0.213	0.25
DASH component 6: Magnesium							
Model 1	−0.018	0.099	0.86	−0.140	0.069	0.056	0.28
Model 2	−0.014	0.103	0.90	−0.130	0.063	0.044	0.26
Model 3	−0.091	0.105	0.99	−0.100	0.060	0.088	0.29
DASH component 7: Calcium							
Model 1	−0.034	0.060	0.57	−0.027	0.049	0.59	0.94
Model 2	−0.040	0.060	0.51	−0.016	0.053	0.76	0.93
Model 3	−0.0263	0.059	0.66	−0.003	0.050	0.94	0.94
DASH component 8: Potassium							
Model 1	−0.016	0.089	0.86	−0.040	0.096	0.68	0.72
Model 2	+0.016	0.095	0.99	−0.019	0.093	0.84	0.67
Model 3	+0.009	0.101	0.92	−0.001	0.086	1.00	0.74
DASH component 9: Sodium							
Model 1	+0.059	0.077	0.45	+0.076	0.085	0.38	0.88
Model 2	+0.065	0.078	0.41	+0.061	0.088	0.50	0.97
Model 3	+0.069	0.076	0.37	+0.069	0.081	0.40	0.91

Abbreviations: DASH—Dietary Approaches to Stop Hypertension; NHANES—National Health and Nutrition Examination Surveys; SEE—standard error of the estimate. Model 1 (age); Model 2 (age, sex, race/ethnicity, marital status, poverty income ratio and education); Model 3 (Model 2 + smoking, alcohol use, physical activity, self-rated health, BMI and ALI). *N*—1922 among men and *N*—2019 among women.

## Supplementary Materials

The following are available online at https://www.mdpi.com/2072-6643/12/5/1510/s1, Supplementary File S1: Sleep Quality Index Variables; Supplementary File S2: Reduced factor analysis and factor loading plot Reduced and rotated factor analysis results with 2-factor extraction: factor loadings/uniqueness (*N* = 3941); Supplementary File S3: DASH diet scoring system; Supplementary File S4: Allostatic Load Score; Table S1: Study sample characteristics by age group, NHANES 2005–2008; Table S2: DASH total score as predictor of sleep quality measures, stratified by age group: multiple linear regression models, NHANES 2005–2008; Table S3. DASH component scores as predictors of sleep quality measures, stratified by age group: multiple linear regression models, NHANES 2005–2008.

## Abbreviations

ALI—Allostatic Load Index; BMI—Body Mass Index; CRP—C- Reactive Protein; DASH—Dietary Approach to Stop Hypertension; DQ—Diet Quality; HEI-2010—Healthy Eating Index, 2010 version; FNDDS—Food and Nutrient Database for Dietary Studies; MEC—Mobile Examination Center; NHANES—National Health and Nutrition Examination Surveys; PIR—Poverty Income Ratio; RDA—Recommended Dietary Allowance; SE—Standard Error; SES—Socio-Economic Status; SRH—Self-Reported Health; USDA—US Department of Agriculture.
