# Supplementary material for: Dietary Approaches to Stop Hypertension (DASH) Score and Its Association with Sleep Quality in a National Survey of Middle-Aged and Older Men and Women
Source: Nutrients. 2020 May 22;12(5):1510. doi: 10.3390/nu12051510 (PMC7284419; doi:10.3390/nu12051510)
Supplement: Supplementary file 1 [file nutrients-12-01510-s001.pdf]

Supplementary File S1. Sleep Quality Index Variables

(**1**) How much sleep do you get (hours): How much sleep do you get (hours)? The range is 1-11 and is truncated at 12 or more. Response of "I don't know" or "refused" were considered missing: **sld010h**

(**2**) How long to fall asleep (min.), 0-50, 60 or more=60: **sld020m**

(**3**) How often do you snore?, 4-point likert scale: 0=never, 1=Rarely (1-2 nights/week), 2= Occasionally (3-4 nights/week), 3=Frequently (5 or more nights/week): **slq030**

(**4**) How often do you snort/stop breathing? 4-point likert scale: 0=never, 1=Rarely (1-2 nights/week), 2= Occasionally (3-4 nights/week), 3=Frequently (5 or more nights/week): **slq040**

(5) How often do you have trouble falling asleep? 5-point likert scale: 0=Never, 1=Rarely (1 time a month), 2=Sometimes (2-4 times a month), 3=Often (5-15 times a month), 4=Almost always (16-30 times a month) : **slq080**

(6) How often do you wake up during the night? 5-point likert scale: 0=Never, 1=Rarely (1 time a month), 2=Sometimes (2-4 times a month), 3=Often (5-15 times a month), 4=Almost always (16-30 times a month) : **slq090**

(7) How often do you wake up too early in the morning? 5-point likert scale: 0=Never, 1=Rarely (1 time a month), 2=Sometimes (2-4 times a month), 3=Often (5-15 times a month), 4=Almost always (16-30 times a month) : **slq100**

(8) How often do you feel unrested during the day? 5-point likert scale: 0=Never, 1=Rarely (1 time a month), 2=Sometimes (2-4 times a month), 3=Often (5-15 times a month), 4=Almost always (16-30 times a month) : **slq110**

(9) How often do you feel overly sleepy during the day? 5-point likert scale: 0=Never, 1=Rarely (1 time a month), 2=Sometimes (2-4 times a month), 3=Often (5-15 times a month), 4=Almost always (16-30 times a month) : **slq120**

(10) How often did you not get enough sleep? 5-point likert scale: 0=Never, 1=Rarely (1 time a month), 2=Sometimes (2-4 times a month), 3=Often (5-15 times a month), 4=Almost always (16-30 times a month) : **slq130**

(11) How often do you take pills to help you sleep? 5-point likert scale: 0=Never, 1=Rarely (1 time a month), 2=Sometimes (2-4 times a month), 3=Often (5-15 times a month), 4=Almost always (16-30 times a month) : **slq140**

(12) How often do you have leg jerks while sleeping? 5-point likert scale: 0=Never, 1=Rarely (1 time a month), 2=Sometimes (2-4 times a month), 3=Often (5-15 times a month), 4=Almost always (16-30 times a month) : **slq150**

(13) How often do you have leg cramps while sleeping? 5-point likert scale: 0=Never, 1=Rarely (1 time a month), 2=Sometimes (2-4 times a month), 3=Often (5-15 times a month), 4=Almost always (16-30 times a month) : **slq160**

(14) Do you have difficulty concentrating when tired? 5-point likert scale: 1=Don't do this activity for other reasons, 2=no difficulty, 3=Yes, a little difficulty, 4=Yes, moderate difficulty, 5=yes, extreme difficulty: **slq170**

(15) Do you have difficulty remembering when tired? 5-point likert scale: 1=Don't do this activity for other reasons, 2=no difficulty, 3=Yes, a little difficulty, 4=Yes, moderate difficulty, 5=yes, extreme difficulty: **slq180**

(16) Do you have difficulty eating when tired? 5-point likert scale: 1=Don't do this activity for other reasons, 2=no difficulty, 3=Yes, a little difficulty, 4=Yes, moderate difficulty, 5=yes, extreme difficulty: **slq190**

(17) Do you have difficulty with a hobby when tired? 5-point likert scale: 1=Don't do this activity for other reasons, 2=no difficulty, 3=Yes, a little difficulty, 4=Yes, moderate difficulty, 5=yes, extreme difficulty: **slq200**

(18) Do you have difficulty getting things done when tired? 5-point likert scale: 1=Don't do this activity for other reasons, 2=no difficulty, 3=Yes, a little difficulty, 4=Yes, moderate difficulty, 5=yes, extreme difficulty: **slq210**

(19) Do you have difficulty with finances when tired? 5-point likert scale: 1=Don't do this activity for other reasons, 2=no difficulty, 3=Yes, a little difficulty, 4=Yes, moderate difficulty, 5=yes, extreme difficulty: **slq220**

(20) Do you have difficulty with work when tired? 5-point likert scale: 1=Don't do this activity for other reasons, 2=no difficulty, 3=Yes, a little difficulty, 4=Yes, moderate difficulty, 5=yes, extreme difficulty: **slq230**

(21) Do you have difficulty using the phone when tired? 5-point likert scale: 1=Don't do this activity for other reasons, 2=no difficulty, 3=Yes, a little difficulty, 4=Yes, moderate difficulty, 5=yes, extreme difficulty: **slq240**

Supplementary File S2. Reduced factor analysis and factor loading plot

Reduced and rotated factor analysis results with 2-factor extraction:

factor loadings/uniqueness (N = 3941)

	**Factor 1**	**Factor 2**	**Uniqueness**
	**Sleepiness and sleep disturbance**	**Poor sleep-related daytime dysfunction**	
sld020m	**0.46**	-0.03	0.79
slq080	**0.71**	0.06	0.49
slq090	**0.75**	0.09	0.44
slq100	**0.66**	0.08	0.56
slq110	**0.63**	0.29	0.51
slq120	**0.60**	0.34	0.53
slq130	**0.63**	0.24	0.54
slq170	0.28	**0.64**	0.51
slq180	0.25	**0.58**	0.60
slq190	0.09	**0.42**	0.81
slq200	0.15	**0.55**	0.67
slq210	0.13	**0.58**	0.64
slq220	0.10	**0.60**	0.63
slq230	0.01	**0.46**	0.79

Bolded factor loadings are >0.40 and considered significant. Factors are labelled based on highest loadings on measured items.

Factor loading plot

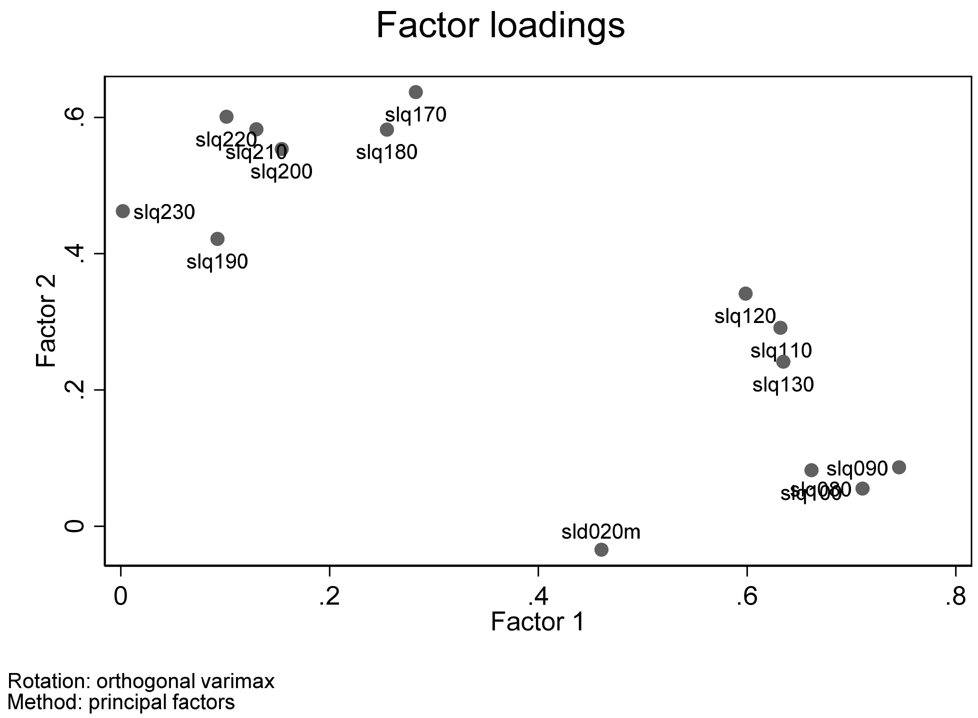

Supplementary File S3. DASH diet scoring system

**Nutrient**	**DASH diet nutrient composition** [a]	**DASH score target (1)**	**Intermediate target (0.5)**
Saturated fat	6% of energy	6% of energy	11% of energy
Total fat	27% of energy	27% of energy	32% of energy
Protein	18% of energy	18% of energy	16.5% of energy
Cholesterol	150 mg	71.4 mg/1000 kcal	107.1 mg/1000 kcal
Fiber	31 g	14.8 g/1000 kcal	9.5 g/1000 kcal
Magnesium	500 mg	238 mg/1000 kcal	158 mg/1000 kcal
Calcium	1240 mg	590 mg/1000 kcal	402 mg/1000 kcal
Potassium	4700 mg	2238 mg/1000 kcal	1534 mg/1000 kcal
Sodium [b]	2400 mg	1143 mg/1000 kcal	1286 mg/1000 kcal

Abbreviation: DASH, Dietary Approaches to Stop Hypertension trial.

[a] Based on a 2100-kcal diet.

[b] Sodium target based on the Sixth Report of the Joint National Committee on Prevention, Detection, Evaluation, and Treatment of High Blood Pressure recommendations.

Source:[1]

1. Mellen PB, Gao SK, Vitolins MZ, Goff DC, Jr. Deteriorating dietary habits among adults with hypertension: DASH dietary accordance, NHANES 1988-1994 and 1999-2004. Archives of internal medicine 2008;168:308-14.

Supplementary File S4. Allostatic Load Score

	**1**	**0.5**	**0**
**SBP**	≥150 mmHg	120 to <150 mmHg	<120 mmHg
**DBP**	≥90 mmHg	80 to <90 mmHg	<80 mmHg
**BMI**	≥30 kg/m^2	25 to <30 kg/m^2	<25 kg/m^2
**HbA1c**	≥6.5%	5.7% to <6.5%	<5.7%
**Total cholesterol**	≥240 mg/dL	200 to <240 mg/dL	<200 mg/dL
**HDL cholesterol**	<40 mg/dL	40 to <60 mg/dL	≥60 mg/dL
**Total/HDL cholesterol ratio**	≥6	5 to <6	<5
**CRP**	≥3 mg/L	1 to <3 mg/L	<1 mg/L
**Albumin**	<3.0 µg/mL	3.0 to <3.8 µg/mL	≥3.8 µg/mL
**Creatinine clearance**	<30 mL/min/1.73 m^2	30 to <60 mL/min/1.73 m^2	≥60 mL/min/1.73 m^2

Sources:[2]

1. Mellen, P.B.; Gao, S.K.; Vitolins, M.Z.; Goff, D.C., Jr. Deteriorating dietary habits among adults with hypertension: DASH dietary accordance, NHANES 1988-1994 and 1999-2004. *Archives of internal medicine* **2008**, *168*, 308-314, doi:10.1001/archinternmed.2007.119.
2. Rodriquez, E.J.; Livaudais-Toman, J.; Gregorich, S.E.; Jackson, J.S.; Napoles, A.M.; Perez-Stable, E.J. Relationships between allostatic load, unhealthy behaviors, and depressive disorder in U.S. adults, 2005-2012 NHANES. *Preventive medicine* **2018**, *110*, 9-15, doi:10.1016/j.ypmed.2018.02.002.

TABLE S1. Study sample characteristics by age group, NHANES 2005-2008

	Age 30-59		Age60+		P_{age}
	Mean/%	(SE)	Mean/%	(SE)	
**Age (years):**					<0.001
Mean ± SEM	44.2	0.3	68.5	0.3	
**Sex:**					<0.001
Male	50.0	0.0	41.1	0.0	
Female	50.0	0.0	58.9	0.0	
**Race/Ethnicity:**					<0.001
Mexican American	7.9	0.01	4.1	0.01	
Other Hispanic	3.8	0.01	2.9	0.01	
Non-Hispanic White	73.3	0.02	83.6	0.02	
Non-Hispanic Black	9.4	0.01	7.1	0.01	
Other	5.6	0.01	2.4	0.01	
**Education:**					<0.001
< Less Than 9th Grade	3.683	0.004	10.693	0.011	
9-11th Grade	10.550	0.012	12.891	0.013	
High School Grad/GED or Equivalent	23.110	0.011	28.489	0.012	
Some College or AA degree	31.252	0.012	23.470	0.015	
College Graduate or above	31.405	0.022	24.457	0.019	
**Marital status:**					<0.001
Married/Living with partner	74.257	0.016	65.823	0.021	
Other	25.743	0.016	34.177	0.021	
**Poverty-income ratio:**					<0.001
< 100%	9.201	0.010	7.071	0.008	
100%-<200%	14.476	0.011	24.701	0.014	
≥ 200%	76.323	0.018	68.228	0.018	
**Smoking status:**					<0.001
Never smoker	54.605	0.013	46.283	0.018	
Ex-smoker	22.371	0.009	41.046	0.018	
Current smoker	23.024	0.014	12.671	0.013	
**Alcohol consumption *(≥ 12 glasses in past 12 months):***					<0.001
Yes	78.695	0.015	67.019	0.019	
No	21.305	0.015	32.980	0.019	
**Physical activity:**					0.025
Yes	45.773	0.026	38.184	0.032	
No	54.227	0.026	61.816	0.032	
**Body mass index (kg/m^2):**					0.35
Mean ± SEM	28.941	0.229	28.671	0.211	
**Self-rated health:**				0.013	
Excellent/Very good/Good	87.801	0.009	84.924		
Fair/Poor	12.199	0.009	15.076		
**Allostatic load**					

Mean ± SEM	2.708	0.041	3.291	0.049	<0.001
**DASH diet total score**					
Mean ± SEM	2.065	0.040	2.494	0.062	<0.001
**DASH diet component 1: Saturated fat**					
Mean ± SEM	0.251	0.009	0.271	0.010	0.089
**DASH diet component 2: Fat**					
Mean ± SEM	0.265	0.008	0.288	0.018	0.051
**DASH diet component 3: Protein**					
Mean ± SEM	0.346	0.012	0.354	0.017	0.65
**DASH diet component 4: Cholesterol**					
Mean ± SEM	0.266	0.009	0.275	0.010	0.54
**DASH diet component 5: Fiber**					
Mean ± SEM	0.143	0.008	0.248	0.013	<0.001
**DASH diet component 6: Magnesium**					
Mean ± SEM	0.177	0.007	0.259	0.015	<0.001
**DASH diet component 7: Calcium**					
Mean ± SEM	0.360	0.010	0.431	0.013	<0.001
**DASH diet component 8: Potassium**					
Mean ± SEM	0.133	0.005	0.239	0.010	<0.001
**DASH diet component 9: Sodium**					
Mean ± SEM	0.122	0.007	0.127	0.013	0.74
**Sleep quality measures**					
**Factor 1: Sleepiness and sleep disturbance**					
Mean ± SEM	0.070	0.024	-.000	0.030	0.095
**Factor 2: Poor sleep-related daytime dysfunction**					
Mean ± SEM	0.084	0.018	-.265	0.031	<0.001

Abbreviations: DASH=Dietary Approaches to Stop Hypertension; nc=not computed; NHANES=National Health and Nutrition Examination Surveys; SEM=Standard Error of the Mean.

N=2,683 among the 30-59y age group, N=1,258 among the 60+ age group.

TABLE S2. DASH total score as predictor of sleep quality measures, stratified by age group: multiple linear regression models, NHANES 2005-2008

	Age30-59<br>N=2,683			Age 60+<br>N=1,258			
	Factor 1: **Sleepiness and sleep disturbance**			*Factor 1:* **Sleepiness and sleep disturbance**			
	β	(SEE)	P_{wald}	β	(SEE)	P_{wald}	$P_{age×DASH}$
Model 1	-0.023	0.017	0.19	-0.028	0.017	0.11	0.72
Model 2	-0.010	0.017	0.56	-0.030	0.018	0.11	0.55
Model 3	-0.007	0.018	0.71	-0.028	0.018	0.13	0.56
	Factor 2: **Poor sleep-related daytime dysfunction**			*Factor 2:* **Poor sleep-related daytime dysfunction**			
	β	(SEE)	P_{wald}	β	(SEE)	P_{wald}	$P_{age×DASH}$
Model 1	-0.023	0.013	0.088	-0.096	0.014	0.51	0.75
Model 2	-0.022	0.013	0.10	-0.005	0.016	0.78	0.78
Model 3	-0.020	0.014	0.18	-0.002	0.016	0.92	0.68

Abbreviations: DASH=Dietary Approaches to Stop Hypertension; NHANES=National Health and Nutrition Examination Surveys ; SEE=Standard Error of the Estimate.

Model 1 (age); Model 2 (age, sex, race/ethnicity, marital status, poverty income ratio and education); Model 3 (Model 2 + smoking, alcohol use, physical activity, self-rated health, BMI and ALI).

TABLE S3. DASH component scores as predictors of sleep quality measures, stratified by age group: multiple linear regression models, NHANES 2005-2008

	Age30-59 *Factor 1* **Sleepiness and sleep disturbance**			Age 60+ *Factor 1* **Sleepiness and sleep disturbance**			
	β	(SEE)	P_{wald}	β	(SEE)	P_{wald}	$P_{age×DASH}$
DASH component 1: Saturated fat							
Model 1	-0.013	0.080	0.13	-0.043	0.012	0.72	0.69
Model 2	-0.057	0.078	0.47	-0.040	0.128	0.76	0.78
Model 3	-0.040	0.079	0.62	-0.073	0.134	0.59	0.96
DASH component 2: Total fat							
Model 1	+0.002	0.059	0.97	-0.012	0.070	0.092	0.085
Model 2	+0.036	0.057	0.53	-0.013	0.067	0.066	0.077
Model 3	+0.044	0.058	0.45	-0.013	0.071	0.068	0.052
DASH component 3: Protein							
Model 1	-0.038	0.041	0.36	-0.010	0.067	0.14	0.50
Model 2	-0.019	0.042	0.65	-0.010	0.067	0.13	0.46
Model 3	-0.023	0.043	0.64	-0.095	0.066	0.16	0.50
DASH component 4: Cholesterol							
Model 1	+0.024	0.046	0.60	+0.018	0.092	0.85	0.89
Model 2	+0.020	0.048	0.69	+0.002	0.086	0.98	0.89
Model 3	+0.018	0.050	0.72	+0.006	0.090	0.95	0.97
DASH component 5: Fiber							
Model 1	-0.205	0.083	0.019	-0.115	0.093	0.226	0.50
Model 2	-0.129	0.086	0.14	-0.110	0.10	0.276	0.69
Model 3	-0.106	0.086	0.23	-0.124	0.11	0.246	0.76

DASH component 6: Magnesium							
Model 1	-0.047	0.080	0.019	-0.101	0.091	0.28	0.69
Model 2	0.007	0.083	0.94	-0.089	0.096	0.36	0.54
Model 3	0.018	0.085	0.83	-0.060	0.096	0.54	0.61
DASH component 7: Calcium							
Model 1	-0.055	0.059	0.33	+0.010	0.081	0.90	0.42
Model 2	-0.050	0.063	0.44	+0.010	0.096	0.91	0.53
Model 3	-0.032	0.061	0.61	0.007	0.086	0.94	0.57
DASH component 8: Potassium							
Model 1	-0.225	0.103	0.036	-0.109	0.098	0.27	0.34
Model 2	-0.211	0.103	0.049	-0.121	0.100	0.23	0.41
Model 3	-0.193	0.061	0.605	-0.095	0.102	0.36	0.33
DASH component 9: Sodium							
Model 1	+0.078	0.074	0.30	-0.031	0.088	0.73	0.20
Model 2	+0.092	0.073	0.21	-0.033	0.089	0.71	0.23
Model 3	+0.085	0.077	0.27	-0.001	0.092	1.00	0.37
	Factor 2 **Poor sleep-related daytime dysfunction**			*Factor 2* **Poor sleep-related daytime dysfunction**			
DASH component 1: Saturated fat	β	(SEE)	P_{wald}	β	(SEE)	P_{wald}	$P_{age \times DASH}$
Model 1	-0.105	0.061	0.095	-0.012	0.068	0.87	0.49
Model 2	-0.103	0.066	0.13	-0.000	0.073	1.00	0.51
Model 3	-0.082	0.070	0.25	-0.014	0.075	0.85	0.69
DASH component 2: Total fat							
Model 1	-0.033	0.043	0.45	-0.067	0.053	0.21	0.50
Model 2	-0.026	0.044	0.56	-0.056	0.053	0.30	0.40
Model 3	-0.020	0.046	0.66	-0.053	0.052	0.31	0.47

DASH component 3: Protein							
Model 1	-0.053	0.057	0.36	-0.043	0.065	0.51	0.76
Model 2	-0.048	0.058	0.41	-0.039	0.064	0.55	0.76
Model 3	-0.062	0.057	0.29	-0.030	0.060	0.62	0.62
DASH component 4: Cholesterol							
Model 1	-0.052	0.042	0.23	-0.071	0.057	0.23	0.64
Model 2	-0.049	0.042	0.26	-0.063	0.053	0.25	0.67
Model 3	-0.049	0.045	0.29	-0.061	0.056	0.28	0.82
DASH component 5: Fiber							
Model 1	-0.168	0.068	0.019	+0.128	0.068	0.067	0.013
Model 2	-0.157	0.067	0.025	+0.143	0.076	0.068	0.020
Model 3	-0.136	0.070	0.062	+0.146	0.081	0.082	0.030
DASH component 6: Magnesium							
Model 1	-0.011	0.075	0.14	-0.0279	0.061	0.65	0.53
Model 2	-0.011	0.075	0.14	-0.0024	0.066	0.97	0.57
Model 3	-0.093	0.076	0.23	+0.001	0.071	0.85	0.57
DASH component 7: Calcium							
Model 1	-0.022	0.042	0.60	-0.0247	0.066	0.71	0.84
Model 2	-0.002	0.043	0.67	-0.0040	0.067	0.95	0.85
Model 3	0.0001	0.043	0.82	-0.0009	0.066	0.99	0.83
DASH component 8: Potassium							
Model 1	-0.039	0.102	0.71	-0.025	0.048	0.64	0.84
Model 2	-0.036	0.100	0.73	-0.002	0.053	0.98	0.87
Model 3	-2.475	0.096	0.80	+0.023	0.053	0.67	0.96
DASH component 9: Sodium							
Model 1	+0.100	0.074	0.18	+0.000	0.068	1.00	0.20
Model 2	+0.087	0.074	0.25	-0.216	0.063	0.97	0.19

Model 3	+0.089	0.072	0.22	1.198	0.060	0.84	0.29

Abbreviations: DASH=Dietary Approaches to Stop Hypertension; NHANES=National Health and Nutrition Examination Surveys; SEE=Standard Error of the Estimate.

Model 1 (age); Model 2 (age, sex, race/ethnicity, marital status, poverty income ratio and education); Model 3 (Model 2 + smoking, alcohol use, physical activity, self-rated health, BMI and ALI). N=2,683 among the 30-59y age group, N=1,258 among the 60+ age group.
